# Patient Perception of Cough in Interstitial Lung Disease; Impact of Cough Hypersensitivity

**DOI:** 10.1007/s00408-024-00723-0

**Published:** 2024-07-08

**Authors:** B. Hirons, K. Rhatigan, L. Wright, H. Kesavan, E. Mackay, P. S. P. Cho, S. S. Birring, K. J. Myall

**Affiliations:** 1 Department of Respiratory Medicine, Chest Unit, Cheyne Wing, King’s College Hospital, Denmark Hill, London, SE5 9RS UK; 2 Centre for Human and Applied Physiological Sciences, King’s College London, London, UK; 3 Action for Pulmonary Fibrosis, Peterborough, UK

**Keywords:** Interstitial lung disease, Cough hypersensitivity syndrome, Quality of life, Impact

## Abstract

**Introduction:**

Cough is common in interstitial lung disease (ILD) and is associated with disease progression, yet its mechanisms are understudied. We investigated cough hypersensitivity features and impact in ILD.

**Methods:**

Participants with ILD and cough (*n* = 195) completed a multiple choice and free text questionnaire on cough sensations/triggers and impacts.

**Results:**

The majority of participants were male (54%), aged > 65 (64%), with idiopathic pulmonary fibrosis (IPF, 75%). Common cough triggers were body position (74%), physical activity (72%), and talking (62%). Common laryngeal sensations were globus (43%), and itch/tickle (42%). Cough impacted everyday life in 55%, and all activities in 31%, causing exhaustion (59%), social embarrassment (70%), urinary incontinence (46% females), and syncope/pre-syncope (12%). The total number of cough-provoking sensations/triggers correlated with impacts; *ρ* = 0.73, *p* < 0.001.

**Conclusion:**

Cough hypersensitivity symptoms are prevalent in ILD and detrimentally affect quality of life. Further studies investigating mechanisms of cough hypersensitivity and targeted pharmacotherapy are warranted.

**Supplementary Information:**

The online version contains supplementary material available at 10.1007/s00408-024-00723-0.

## Introduction

Chronic cough (CC, lasting > 8 weeks) is a condition affecting approximately 10% of the global population and is associated with significant impact on quality of life [1]. Cough hypersensitivity is hypothesised as a key mechanistic driver of CC, and is mediated through sensitisation of one or both of peripheral and central neuropathways [2]. Clinical features suggestive of cough hypersensitivity are akin to neuropathic pain, and include allotussia (cough triggered by non-tussive stimuli, e.g. talking), hypertussia (excessive cough to known tussive stimuli, e.g. aerosols), and laryngeal paraesthesia (e.g. throat tickle) [3].

Interstitial lung disease (ILD) is an umbrella term for a diverse set of diseases causing inflammation and fibrosis within the extracellular matrix of the lung. There are over 200 aetiologies of ILD, of which idiopathic pulmonary fibrosis (IPF) is the most common, accounting for up to 39% [4]. CC is common in ILD, particularly IPF, and affects 50–90% of patients [5–8]. The mechanism of cough in ILD is poorly understood, and may involve both inflammation and mechanical distortion of the airways which may cause sensitisation and stimulation of chemoreceptors and mechanoreceptors, respectively [7]. CC in ILD substantially impacts quality of life [9, 10], and the presence of CC is associated with worse outcomes including disease progression and mortality [8, 11, 12]. Few effective treatment options are available for CC in ILD [6, 7, 13]; however, recent separate randomised controlled trials have reported efficacy with morphine and nalbuphine (an opioid agonist–antagonist) in IPF-associated cough [14, 15]. Despite the significant impact of CC in ILD, the different phenotypes of CC are understudied.

Phenotyping cough in ILD will aid understanding of cough endotypes leading to improved drug development and personalised care for patients. This study aimed to conceptualise cough hypersensitivity features and impacts in ILD from the patients’ perspective.

## Methods

### Protocol

Patients with ILD and cough were invited by Action for Pulmonary Fibrosis (APF; registered England and Wales charity number 1152399) to complete an anonymous online questionnaire. Responses were excluded from analysis if the patient reported no cough (*n* = 2) or did not select an ILD diagnosis (*n* = 14).

### Questionnaire

The online questionnaire was designed in collaboration with APF for patients with ILD with self-identified cough. It aimed to elicit common cough triggers, sensations, and impacts on quality of life with mixed methods through multiple choice and free text responses. A binary scale was used to detect the presence of 10 cough triggers and 3 laryngeal sensations. The impact of cough was assessed across four domains: frequency of impact, 7 daily activities, 7 physical impacts, and 4 emotional impacts. Qualitative questions were “How does the cough make you feel?”, “What is the biggest impact of your cough?”, and “Have you found ways to deal with your cough?”. A copy of the questionnaire is available in the Online Supplement, Table E2.

### Statistical Analysis

Normality was assessed with D’Agostino-Pearson test. Non-parametric continuous variables were expressed as median (interquartile range) and categorical variables as proportions. Non-parametric unpaired data were evaluated by the Mann–Whitney U test, and correlations between variables were analysed using the Spearman’s rank correlation coefficient (*ρ*). All statistical analyses were performed using Prism^®^ Version 10.0.1 (GraphPad Software, San Diego, California, USA). Qualitative data were analysed by open coding through thematic analysis as described by Willis et al. [16]. Initial analysis was done by first author (BH) and reviewed with co-author (SB) and senior author (KM).

## Results

### Participants

A total of 195 participants with ILD and cough were included; 125 (64%) aged > 65 years, 89 (46%) female sex, 186 (96%) white/Caucasian (supplementary table E1). Aetiology of ILD was IPF in 147 (75%) participants; connective tissue disease (CTD)-associated in 13 (7%); chronic hypersensitivity pneumonitis (CHP) in 12 (6%); nonspecific interstitial pneumonia (NSIP) in 5 (3%); sarcoidosis in 1 (0.5%); drug-induced in 1 (0.5%); and unspecified in 16 (8%).

### Triggers and Laryngeal Sensations

**Fig. 1:**
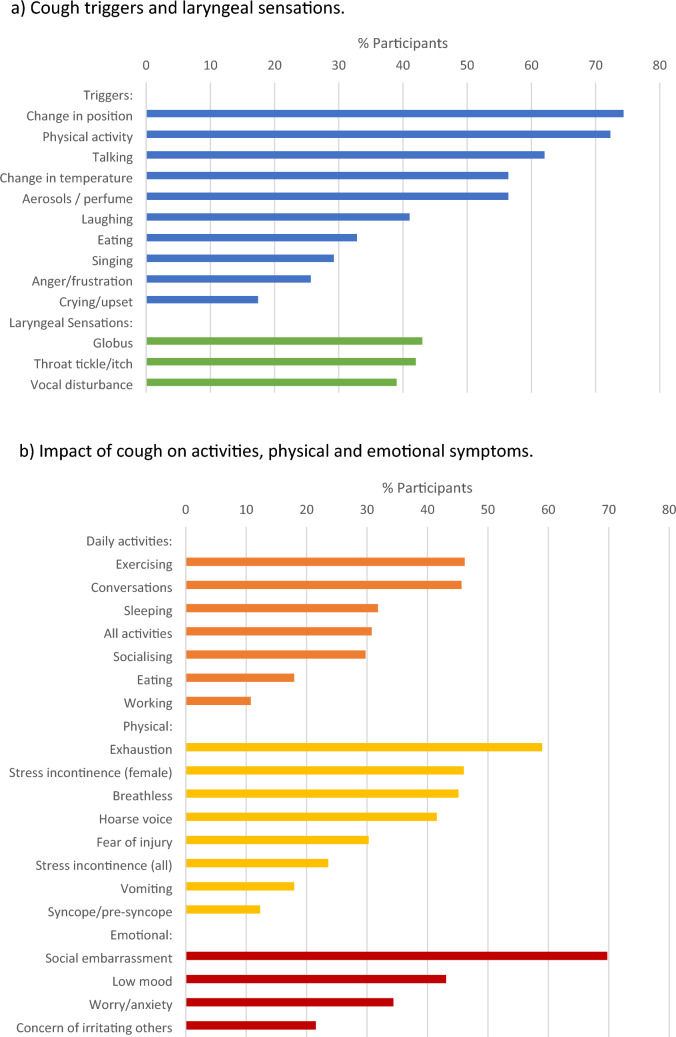
**a**, **b** Cough triggers, sensations, and impacts in participants with ILD (*n* = 195).

Cough triggers and laryngeal sensations were common in ILD patients with cough; the median (IQR) number of triggers reported by participants was 5 (3–7) and laryngeal sensations was 1 (1–2) (Fig. 1). The most common triggers were change in body position (*n* = 145; 74%), physical activity (*n* = 141; 72%), and talking (*n* = 122; 62%). Laryngeal symptoms: globus, itch/tickle, and voice disturbance were reported by 84 (43%), 82 (42%), and 77 (39%) participants, respectively.

### Impact of Cough

The majority of participants reported that cough impacted their life every day (*n* = 108; 55%) (Fig. 1), and 60 (31%) participants reported that cough affected all their daily activities. The total number of impacts reported by the survey was 18. The most commonly affected activities were exercise and conversations; reported by 90 (46%) and 89 (46%) participants, respectively. Multiple physical impacts from cough were reported; the most common were exhaustion (*n* = 115; 59%) and breathlessness (*n* = 88; 45%). Stress incontinence was common in female participants (*n* = 41; 46%). Cough syncope or presyncope was reported by 24 (12%) participants. The most common emotional impacts from cough were social embarrassment (*n* = 136; 70%) and low mood (*n* = 84; 43%).

### Relationship Between Cough Triggers, Sensations, and Impacts

**Fig. 2:**
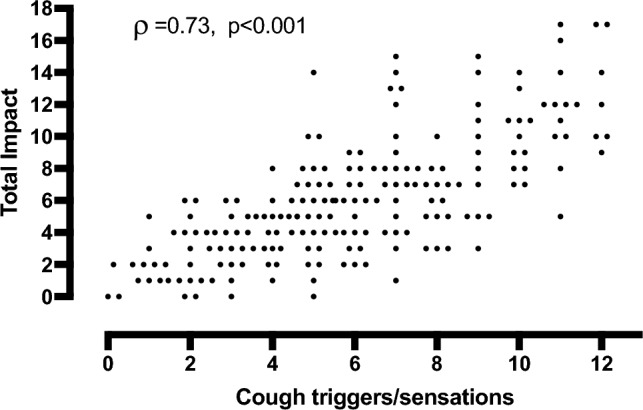
The association between the total number of cough-provoking triggers/sensations (range 0–13) and impacts (range 0–18)

The number of reported triggers and sensations (range 0–13) demonstrated a strong correlation with the number of impacts (range 0–18) (*ρ* = 0.73, *p* < 0.001) (Fig. 2). Furthermore, the number of triggers correlated strongly with impacts (*ρ* = 0.71) and moderately with sensations (*ρ* = 0.32) (both *p* < 0.001). The number of sensations also correlated moderately with impacts (*ρ* = 0.40, *p* < 0.001). The total number of triggers and sensations was higher in patients whose lives were impacted by cough every day or most days (*n* = 170) than those occasionally or never affected (*n* = 25); median (IQR) 6 (4–9) vs 4 (3–5) (*p* < 0.001).

### Qualitative Analysis of the Question “How Does the Cough Make You Feel?”

All participants answered the question “How does the cough make you feel?” (Table 1). Seven themes were elicited, and their corresponding frequencies were: low energy (44%), worried (24%), embarrassed (23%), frustrated (21%), breathless (17%), low mood (11%), and discomfort (5%). Illustrative examples (Table 1) included,

The cough can make me completely exhausted and temporarily unable to carry out simplest of function.

It makes me feel like I’m dying and can’t breathe.

**Table 1:** Codes and themes elicited from answers to “How does the cough make you feel?”, in participants with ILD (*n* = 195)

Themes	*n* (%)	Codes elicited	Illustrative quotes to question "how does the cough make you feel?"
Low energy	87 (44)	Exhausted/tired/drained/weary/fatigue/low energy/worn out	“The cough can make me completely exhausted and temporarily unable to carry out simplest of function”
Worried	48 (24)	Stress/anxious/nervous/worry/frightened/scared/panic/distress/desperate	“Totally drained and stressed and panicky that I will not be able to breathe”
Embarrassed	46 (23)	Embarrassed/self-conscious/judged	“Visible sign of my illness, embarrassing”
Frustrated	41 (21)	Annoyed/frustrated/irritated/nuisance	“Frustrated, inhibiting, restricting”
Breathless	34 (17)	Breathless/can’t breathe/need air	“It makes me feel like I’m dying and can’t breathe”
Low mood	22 (11)	Depressed/low in mood/lifeless/hopeless/struggle/fed up/isolated	“Not wanting to be here i.e. just wanting to die”
Discomfort	10 (5)	Pain/uncomfortable/discomfort/hurt/choke	“Breathless, painful ribs, damaged vocal chords, low mood”

## Discussion

To our knowledge, this is the largest study to investigate patient reported features of cough hypersensitivity in ILD. Furthermore, we are the first to report a relationship between the number of cough hypersensitivity features and health status impact. Qualitative analysis lent weight to the findings that cough severely impacts the quality of life in patients with ILD.

Here, participants with ILD reported many features consistent with cough hypersensitivity (CH), thus suggesting that CH may be a phenotype of cough in ILD and play a mechanistic role [1]. Whilst few studies have investigated CH in ILD comprehensively, multiple cough triggers and heightened cough reflex sensitivity have been reported in IPF, CT-ILD, and sarcoidosis [7, 17]. Neurally mediated CH is hypothesised as a key mechanism in patients with CC that is refractory to treatment [1, 2]. CH in ILD remains enigmatic, and may involve both peripheral and central neural pathways. Inflammation and architectural distortion by fibrosis may sensitise peripheral airway chemoreceptors and mechanoreceptors [7]. However, a peripherally acting P2X2/3 receptor antagonist (Gefapixant) lacked efficacy in IPF associated cough despite positive RCTs in refractory chronic cough [18]. Conversely, recent multi-centre trials with opioids (morphine and nalbuphine) reported efficacy in IPF-associated cough [14, 15]. Taken together, the neuropathophysiology of chronic cough in ILD may be more centrally located. Indeed, recent functional neuroimaging and physiological studies have identified central mechanisms of cough in refractory chronic cough [19].

Several studies have detailed the impact of cough in ILD [9, 10], including an association between cough and disease severity [8, 11, 12]. We report that self-reported CH features were associated with the impact on quality of life. Whilst our data does not indicate causality, future development of CH-targeted therapy may have a direct impact on quality of life in ILD-associated chronic cough.

Our study has certain limitations. Selection bias may be present due to the recruitment of participants through a patient-driven charity, and the use of an online questionnaire could have excluded under-served cohorts who are not digitally capable or who do not understand English. The lists of cough hypersensitivity features and impacts were not exhaustive to avoid response fatigue; these could be developed for further study with patient input. Furthermore, the lack of validated patient reported outcome measures impedes interpretation and direct comparison with previous studies. Future studies could include use of standardised hypersensitivity questionnaires such as the Hull Cough Reflux Questionnaire (HARQ) or Cough Hypersensitivity Questionnaire (CHQ). The questionnaire did not assess the subjective severity of cough and, as it was anonymous, objective measures of cough or ILD severity were unavailable. Comorbidities associated with CC (e.g. gastroesophageal reflux disease [GORD], asthma, rhinitis, bronchiectasis) are common in ILD, and may contribute to cough hypersensitivity [11, 20]. This was not assessed in our study; however, empiric treatment of these conditions, such as PPI for acid-GORD, does not benefit cough [10, 11]. Finally, as with all questionnaires, the presence of recall bias cannot be excluded, such as with the self-reported underlying ILD diagnosis.

## Conclusion

The cough trigger and sensation profile in ILD is suggestive of a cough hypersensitivity phenotype, and is associated with worse patient reported impacts across multiple domains. For patients with ILD, a focus on symptoms and quality of life is imperative given the lack of treatment options which alter the disease course. Further studies should investigate the phenotypes and endotypes of cough hypersensitivity in ILD as well as appropriate cough hypersensitivity pharmacotherapy.

### Supplementary Information

Below is the link to the electronic supplementary material.

Supplementary file1 (DOCX 15 KB)

## Data Availability

The authors confirm that the data supporting the findings of this study are available within the article.
