# Supplementary material for: Patient Perception of Cough in Interstitial Lung Disease; Impact of Cough Hypersensitivity
Source: Lung. 2024 Jul 8;202(4):425–30. doi: 10.1007/s00408-024-00723-0 (PMC11272731; doi:10.1007/s00408-024-00723-0)
Supplement: Supplementary file 1 — Supplementary file1 (DOCX 15 KB) [file 408_2024_723_MOESM1_ESM.docx]

## Online Supplement

Supplementary Table E1. Patient demographics

| **Age** |  |
| --- | --- |
| 36 - 55 | 17(9) |
| 56 - 65 | 54(28) |
| 66 - 75 | 63(32) |
| >76 | 60(31) |
| **Female** | 89(46) |
| **Ethnicity:**  White  Asian  Other/unspecified | 184(94)  4 (2)  7 (4) |
| **ILD diagnosis** |  |
| IPF | 147(75) |
| CT-ILD | 13(7) |
| CHP | 12(6) |
| NSIP | 5(3) |
| Sarcoidosis | 1(1) |
| Drug-induced | 1(1) |
| Unspecified | 16(8) |

Data displayed as n(%)

Supplementary Table E2. Survey questions completed by participants with interstitial lung disease (ILD) and persistent cough.

| **Multiple choice questions:** |  |
| --- | --- |
| What is your age range? (years) | 36-45,  46-55,  56-65,  66-75,  76 and over |
| How would you define your gender? | Male,  Female,  Would rather not say,  Other (please specify) |
| What is your ethnicity? | Asian,  Black,  Latino,  White,  Other (please specify) |
| Please select your underlying condition | Chronic Hypersensitivity Pneumonitis (CHP),  Connective Tissue Disease ILD (CTD-ILD),  Drug induced ILD,  Idiopathic Pulmonary Fibrosis (IPF),  Non-specific interstitial pneumonia (NSIP),  Sarcoid,  unspecified ILD (uILD),  Other (please specify) |
| How frequently does your cough affect your everyday life? | Every day,  most days,  occasional days,  never |
| Does the following trigger your cough? | Change in position,  physical activity,  talking,  change in temperature,  aerosols or perfume,  laughing,  eating,  singing,  anger or frustration,  crying or upset,  other (please specify) |
| How would you describe your cough? | Persistent tickle / itch in throat,  feeling of restriction/blockage in throat (globus),  hoarse voice,  other (please specify) |
| Does the cough regularly stop you doing any of the following? | Affects all my daily activities,  exercising,  conversations,  sleeping,  socialising,  eating,  working |
| Do you regularly experience any of the following due to your cough? | Exhaustion,  stress incontinence,  breathlessness,  hoarse voice,  fear of hurting myself e.g. back or ribs,  vomiting,  cough syncope or makes me feel faint,  social embarrassment,  low mood or depression,  worry or anxiety,  concern of irritating others,  Other (please specify) |
| **Free text questions:** |  |
| How does the cough make you feel? | |
| What is the biggest impact that your cough has on you? | |
| Have you found ways to deal with your cough? | |
